# Comparison of the Olfactory Bulb Volume and the Olfactory Tract Length Between Patients Diagnosed with Essential Tremor and Healthy Controls: Findings in Favor of Neurodegeneration

**DOI:** 10.7759/cureus.5846

**Published:** 2019-10-06

**Authors:** Erman Altunisik, Ali H Baykan

**Affiliations:** 1 Neurology, Adiyaman University Faculty of Medicine, Adiyaman, TUR; 2 Radiology, Adiyaman University Faculty of Medicine, Adiyaman, TUR

**Keywords:** essential tremor, magnetic resonance imaging, olfactory bulb volume, olfactory tract length

## Abstract

Purpose

Essential tremor (ET) is the most common movement disorder. In recent years, an increasing number of studies have shown that this disease also has a variety of non-motor findings and may be of a neurodegenerative nature. This study aimed to evaluate the olfactory bulb volume (OBV) and the olfactory tract length (OTL) and to demonstrate possible neurodegeneration in ET patients using magnetic resonance imaging (MRI).

Methods

The study included 30 ET patients (mean age=29.53±11.82 years) and 30 healthy controls (mean age=30.00±11.68 years). In the cranial MRI examination of both groups, the right, left and total OBV values ​​were measured in mm^3^ and the right and left OTL values ​​were calculated manually in mm.

Results

There was no significant difference between the patient and control groups in the measured OBV values, but the OTL value of the patient group was statistically significantly lower than the control group.

Conclusion

Our study showed that the olfactory system might be involved in ET cases. We think that olfactory dysfunction, one of the non-motor symptoms in ET, can be clearly elucidated through both anatomical and functional studies, to be conducted with larger patient groups.

## Introduction

Essential tremor (ET) is globally the most common movement disorder characterized by a postural and/or kinetic tremor. It is characterized by an 8-12 Hz kinetic tremor that occurs during voluntary movement, which may later be accompanied by head and voice tremors [1]. The prevalence of ET in all age groups is 0.9%, which increases to 4.6% in individuals aged over 65 years [2]. In Turkey, the prevalence of ET has been reported as 4% [3]. Despite the high prevalence of the disease, its pathogenesis remains unknown. ET has been defined as a monosymptomatic, slow-progressing, benign motor system disease characterized by a tremor during voluntary movement, but an increasing number of studies in recent years have shown that this disease also has non-motor findings and may be of a neurodegenerative nature [1]. In addition to motor findings, such as tremor, bradykinesia (mild), and cerebellar dysfunction, ET is increasingly described as having non-motor findings, including postural instability, loss of smell and hearing ability, mild cognitive impairment, neuropsychiatric symptoms, and sleep disorders [4]. This wide range of symptoms, with increased prevalence with age and the progressive course of the disease, supports the idea that ET has a neurodegenerative nature [5].

The olfactory bulb (bulbus olfactorius) is a bulging extension of the olfactory tract [6] located in the olfactory sulcus on the inferior side of the frontal lobe. It has a long, oval, and flat neural structure and is linked to the brain by the olfactory tract [7]. Olfactory degeneration may be associated with structural and/or functional changes in various neurodegenerative diseases such as Parkinson’s disease, Alzheimer’s disease, Huntington’s disease, and motor neuron disease [8].

The identification of the olfactory system on magnetic resonance imaging (MRI) was first undertaken by Suzuki et al. [9]. Later, Yousem et al. [10] developed the standard olfactory bulb volume (OBV) measurement method to be used in MRI studies. In many of the following studies, MRI was shown to be a reliable method for the evaluation of the olfactory system [11]. The measurement of OBV using MRI has been the subject of research on neurological, neurodegenerative, psychiatric, and various other diseases.

Based on the recent literature suggesting that ET does not have a monosymptomatic nature and can present with a neurodegenerative clinical picture, in this study, we aimed to show the possible neurodegeneration in ET using MRI examination and OBV and olfactory tract length (OTL) measurements.

## Materials and methods

Ethics approval was obtained from the Ethics Committee for the study. Informed consent was not obtained from parents, as the study was retrospective.

This retrospective study included patients under 60 years of age, who presented to the general neurology polyclinic of our hospital between January and June 2019, were diagnosed according to the consensus statement on the classification of tremors 2018 [12], and underwent cranial MRI for differential diagnoses. Routine hemogram, biochemistry, and hormone analyses (thyroid hormones, vitamin B12, folic acid, liver and kidney tests, acute phase reactants, sedimentation rate, etc.) were performed for all patients. Individuals who were found to have any disorder that might cause tremors and those who had a history of alcohol abuse or excessive coffee consumption were excluded from the study. Furthermore, patients using antipsychotic, antiepileptic, antidepressant, or antiarrhythmic drugs were also not included in the study. It was determined whether any of the cases had Kayser-Fleischer rings to exclude Wilson’s disease. Patients with chronic and/or systemic diseases (diabetes, hypertension, hyperlipidemia, etc.), history of malignancy, or family history of a neurodegenerative disease were excluded from the study. Also, patients with head tremor and dystonic tremor were excluded from the study. The control group was formed with individuals who, in addition to meeting the exclusion criteria, did not have a history of tremor or a family history of tremor, applied to the polyclinic for non-degenerative reasons, such as headache, vertigo, and tinnitus, and underwent cranial MRI. The sociodemographic characteristics of both groups, such as age and gender, were recorded.

MRI protocol

All cases with normal reports for non-enhanced brain MRI and good MRI quality were included in the study. The brain MRI scans were re-evaluated by a radiologist with more than 10 years’ experience in brain MRI.

All studies were performed using a 1.5 T system (Achieva; Philips Medical Systems, Best, The Netherlands) using a head coil. Contrast material was not used. These images were utilized for volumetric and morphometric measurements. The B-FFE 3D T2-weighted images were obtained in the coronal plane (repetition time (TR), 6.5 ms; echo time (TE), 3.4 ms; field-of-view (FOV) 180 x 180 mm; no significant abnormality (NSA), 2; thickness, 1 mm; GAP, -0.5 mm; slice, 75; matrix, 308 x 308 mm) and 3D T1-weighted images in the sagittal plane (TR, 8.2 ms; TE, 4.0 ms; FOV, 140 x 156; NSA, 4; thickness, 1,2 mm; GAP, -0.5 mm; slices, 40; matrix 252 x 278 mm).

Image analysis

The bilateral volume of the olfactory bulb (OB) and OTL was measured in all subjects. OB was observed as a hypointense ovoid structure surrounded by a hyperintense cerebrospinal fluid on T2-weighted series. The sections were angulated perpendicular to the cribriform plate.

**Figure 1:**
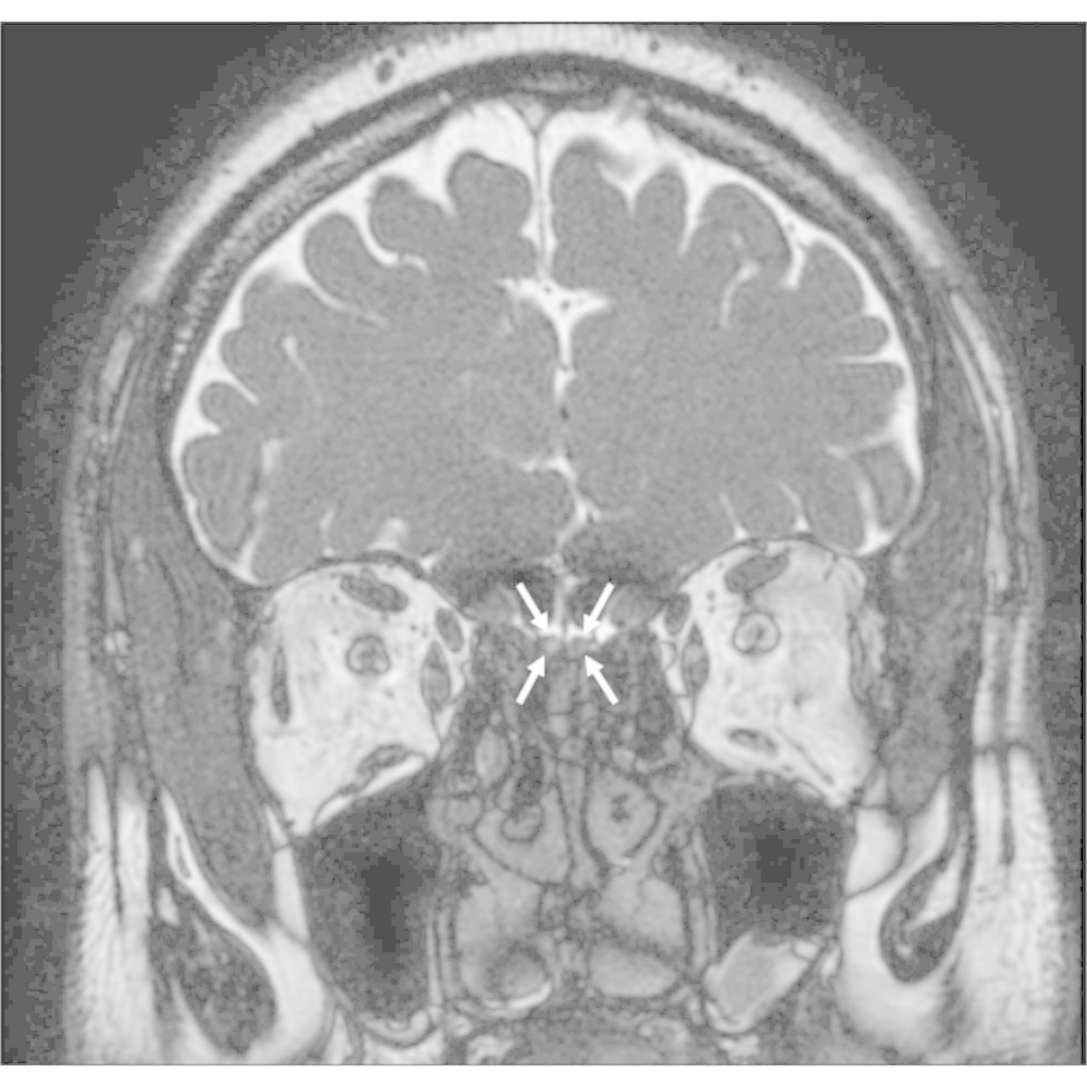
Coronal T2-weighted image Coronal T2-weighted images showing the OBs on both sides with a hypointense ovoid structure (arrow). Note the hyperintense CSF surrounding the OBs. OB: olfactory bulb; CSF: cerebrospinal fluid

**Figure 2:**
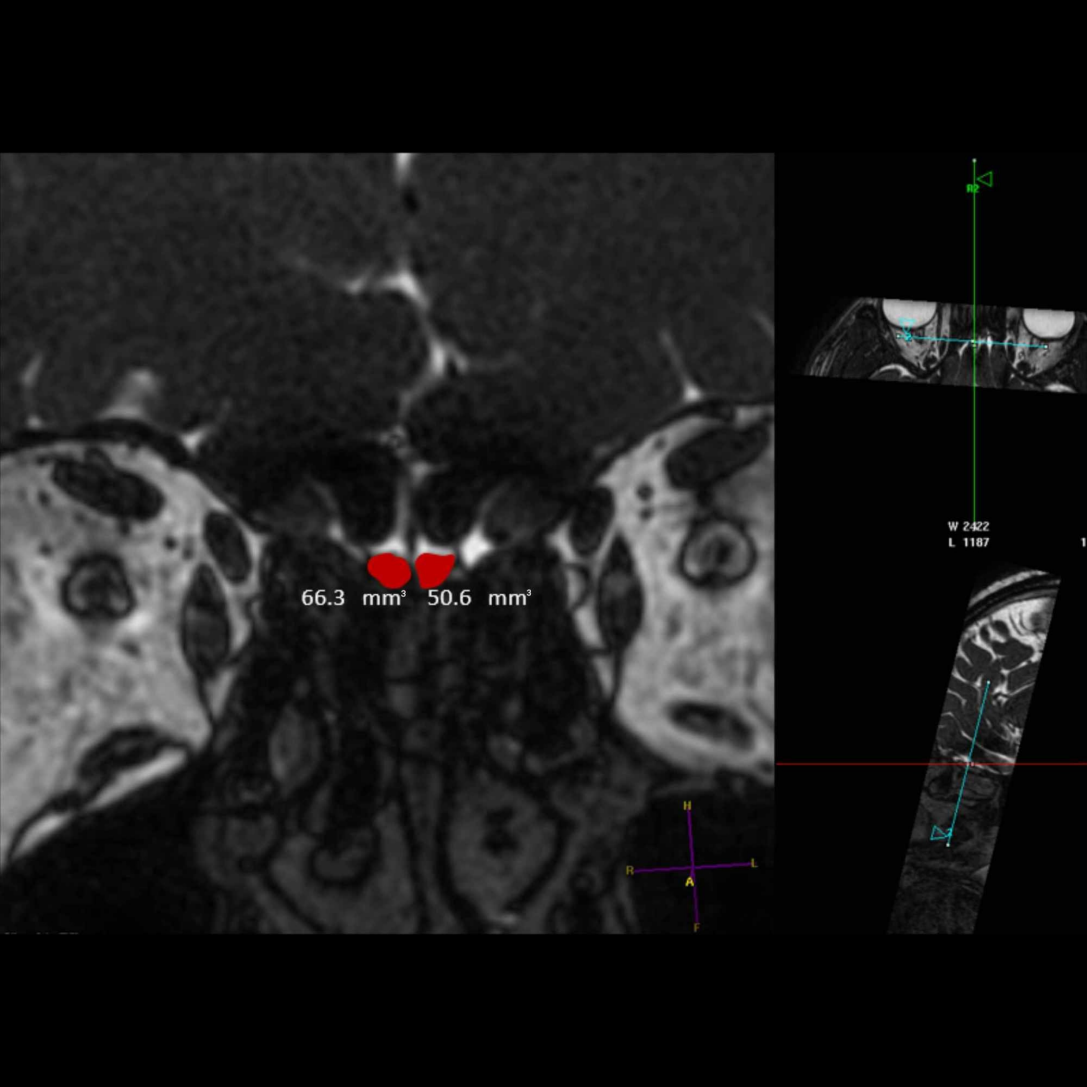
Coronal T2-weighted image Semi-automatic measurement of OBV on the MPR 3D workstation using manual segmentation. To minimize the possibility of errors in volumetric measurements, the images were magnified as much as possible. Both OBs are shown in red and their measured volumes are given underneath in mm^3^. OB: olfactory bulb, OBV: olfactory bulb volume; MPR: multiplanar reformation; 3D: three-dimensional

The volume measurements were performed by an experienced radiologist using a multiplanar reformation (MPR) view 3D workstation by manual segmentation and semi-automatically based on the contour stack principle OBV was calculated as mm^3^ (Figures 1-2).

**Figure 3:**
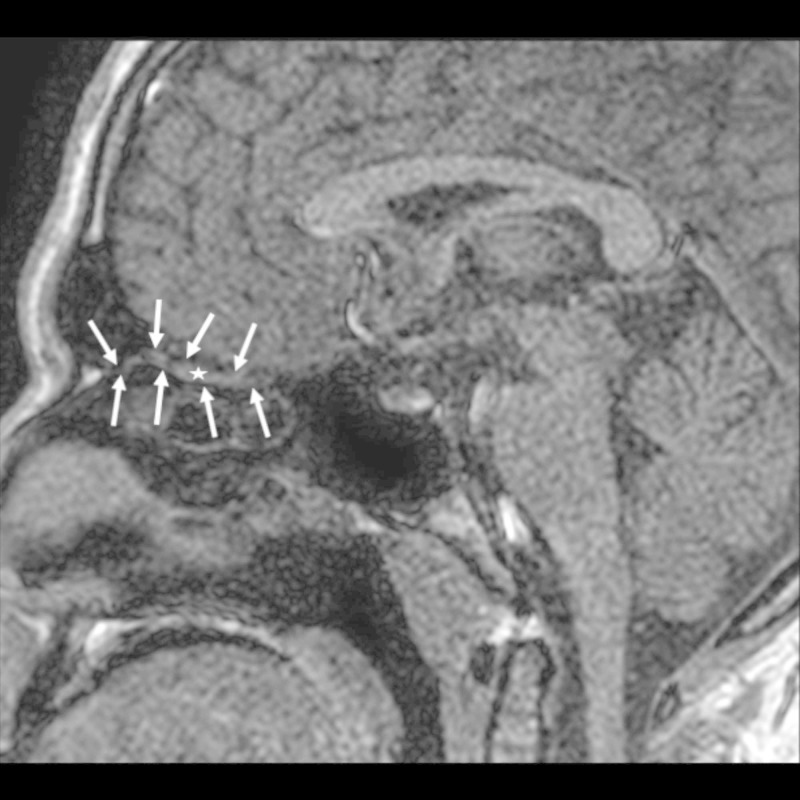
Sagittal T1-weighted image Sagittal T1-weighted image showing the boundaries of the left olfactory tract (arrows). The midsection of the olfactory tract is indicated by an asterisk.

All the OTL measurements were undertaken by an individual who was blinded to the diagnoses of the patients. OTL was measured on sagittal 3D T1-weighted images at the section where the nerve tracing was best visualized, and the values were recorded in mm (Figure 3).

Statistical analysis

Statistical analysis was performed using SPSS program version 17.0 (IBM Corp., Armonk, NY, US). The normality of the distribution of variables was examined by histogram graphs and the Kolmogorov-Smirnov test. The descriptive analyses were expressed as mean and standard deviation and median and minimum-maximum values. A comparison of the normally distributed (parametrical) variables between the groups was undertaken using the independent t-test while non-parametric variables with a non-normal distribution were compared using the Mann-Whitney U test. The Spearman correlation test was performed to determine the correlations between the measurement data. P-values ​​below 0.05 were accepted as statistically significant.

## Results

A total of 60 individuals, 30 patients with ET and 30 healthy controls, were included in this study. Twenty-six (43.33%) were male and 34 (56.67%) were female. The patient group consisted of 17 female and 13 male patients. The control group contained 19 women and 11 men. The mean age of all individuals was 29.77±11.65 years (Table 1). The mean age was calculated as 29.53±11.82 years for the patient group and 30.00±11.68 years for the control group. The mean ages of the male and female populations were determined as 29.04±10.68 and 30.32±12.47 years, respectively. The socio-demographic characteristics, such as the female-male ratio, education level, and marital status were similar in both groups.

**Table 1:** Comparison of the measurements between patient and control groups OBV: olfactory bulb volume (OBV); OTL: olfactory tract length

	Group						p¹
	Control			Patient			
	Mean	SD	Median	Mean	SD	Median	
Right OBV	54.20	±21.02	52.10	44.51	±21.55	40.55	0.083
Left OBV	54.99	±23.05	52.85	44.75	±28.27	40.90	0.129
Total OBV	109.19	±40.03	115.25	89.26	±46.51	82.70	0.089
Right OTL	24.61	±5.48	24.25	20.06	±5.73	19.05	0.003
Left OTL	23.93	±4.57	24.45	19.59	±4.16	19.30	<0.001

In the male population, the right OBV value was 54.25±21.25 mm^3^, left OBV 55.77±25.56 mm^3^,^ ^and total OBV 110.02 ±43.39 mm^3^, and among the females, these parameters were measured as 45.63±21.53 mm^3^,45.36±25.96 mm^3^and 90.96 ±43.61 mm^3^, respectively. The right and left OTL values were determined as 23.38±5.70 mm and 22.47±5.44 mm, respectively, in men, and 21.53±6.20 mm and 21.21±4.36 mm, respectively, in women. There was no statistically significant difference between males and females in the comparison of the OBV and OTL values according to gender (Table 2).

**Table 2:** Comparison of measurements according to gender OBV: olfactory bulb volume; OTL: olfactory tract length

	Gender						p¹
	Male			Female			
	Mean	SD	Median	Mean	SD	Median	
Right OBV	54.25	±21.25	52.10	45.61	±21.53	40.85	0.127
Left OBV	55.77	±25.56	53.05	45.36	±25.96	38.65	0.127
Total OBV	110.02	±43.39	117.75	90.96	±43.61	82.50	0.063²
Right OTL	23.38	±5.70	23.40	21.53	±6.20	20.35	0.241
Left OTL	22.47	±5.44	22.95	21.21	±4.36	21.65	0.321

When the measured OBV values were analyzed according to the group of individuals, the right, left, and total OBVs were found to be 44.51±21.55 mm^3^, 44.75±21.55 mm^3^, and 89.26±46.51 mm^3^, respectively, for the patient group, and 54.20±21.02 mm^3^, 54.99±23.05 mm^3^, and 109.19±40.03 mm^3^, respectively, for the control group. There was no statistically significant difference between the patient and control groups in terms of the three OBV parameters. Concerning the OTL measurements, the right OTL was 20.06±5.73 mm and the left OTL was 19.59±4.16 mm in the patient group, whereas the control group had a right OTL of 24.61±5.48 mm and a left OTL of 23.93±4.57 mm. Both right and left OTL values were statistically significantly lower in the patient group as compared to the controls (p =0.003; p <0.001) (Table 3).

**Table 3:** Diagnosis, gender, and age of participants

		N	%
Group	Control	30	(50.00)
	Patient	30	(50.00)
Gender	Male	26	(43.33)
	Female	34	(56.67)
Age*		29.77 ± 11.65	26.50 (18.00 - 56.00)

## Discussion

In recent years, the growing number of studies on ET have increased the range of symptoms of the disease, making it far from being monosymptomatic, which used to be part of its definition [13-14]. Research, focusing on cognitive deficits, neuropsychiatric symptoms, and neuroimaging studies in ET patients, has raised the question of whether ET is a benign disease or a motor sign of a neurogenic process reflected in the clinical picture. In light of this information, we decided to conduct this study with a neuroanatomical basis to determine whether ET affects the olfactory system, which is a non-motor finding in ET patients. To the best of our knowledge, this is the first study in the literature to examine the OBV and OTL values ​​in order to demonstrate the neurodegenerative nature of ET.

Different and controversial results have been obtained from a number of studies investigating olfactory dysfunction in ET patients. In one of these studies, the smell identification test was administered to the patients with ET and the controls, and the test scores were found to be significantly lower in the patient group as compared to the control group [15]. In another study investigating non-motor symptoms in 121 ET cases and 54 patients that developed idiopathic Parkinson’s disease (IPD) after ET, olfactory dysfunction was reported in 54.6% and 83.3%, respectively [16]. However, there is also research indicating no statistically significant difference in olfactory dysfunction between the ET patients and the controls [17]. While these studies mostly investigated the functional effect of the olfactory system, we focused more on the neuroanatomical aspects of the disease, contributing a different perspective to the literature.

Our review of the literature revealed that OBV has been most frequently investigated in IPD. In their study evaluating 29 patients with IPD, Wang et al. reported that both OBV and olfactory sulcus (OS) depth were significantly lower than healthy controls [18]. In another work comparing the results of six studies conducted in China with a total of 216 IPD cases, it was found that OBV was significantly decreased in IPD patients in three of these studies, while no significant difference was observed in two studies. In the last study, OBV was higher in IPD patients than in the control group [19]. Investigation of whether there is a relationship between IPH and ET, both having the cardinal finding of tremor, has been the subject of many studies. Barbeau and Pourcher [20] noted a significant family history of ET in patients with early-onset Parkinson’s disease. Similarly, Roy et al. [21] found a high rate of ET in the families of patients with Parkinson’s disease, which also has an onset symptom of tremor. According to these results, it can be considered that IPD and ET have similar genetic and neuroanatomical bases, which raises the question of whether there are also similarities between the two diseases about olfactory dysfunction. As revealed by the findings mentioned here, the olfactory system has been extensively investigated in IPD patients, and both functional and neuroanatomical effects on this system have been demonstrated. The similar genetic and neuroanatomical basis of the two diseases is one of the factors that led us to select our patient group from ET cases.

In this study, no significant difference was found between the right, left, and total OBV values ​​in the comparison of patient and control groups (p = 0.083, 0.129 and 0.089, respectively). Due to the ovoid structure of the OB, the anterior part is generally easier to evaluate than the posterior part. This can explain the conflicting results obtained from different studies [22]. In the current study, the measurement of the OBV value was performed with difficulty, and the measurement, undertaken manually using a workstation, took approximately 20 minutes for each patient. The difficulties involved in this method and the long measurement time indicate that in clinical practice, MRI measurement of OBV is not feasible for clinicians. An important feature of OB is its plasticity. In animal studies, the OB showed a high degree of plasticity even in adult subjects [23]. This plasticity can also explain why some studies showed no significant difference in the OBV value in patients with neurodegenerative diseases as compared to the control group or even revealed higher values in the former. Although it is a usable method, there is a wide OBV variation among individuals. While the OBV of men is larger than that of women, the mean decrease with age is equal in both sexes [24]. The individuals in the patient and control groups included in our study were selected from those aged below 60 years to eliminate the effect of reduction in OBV values with age. In addition, we did not find any significant difference in OBV between women and men. Volumetric studies performed in healthy individuals previously provided the standard normal ranges ​​ as 41-97 mm^3^ for the right OBV and 37-98 mm^3^ for the left OBV [22]. This proves that OBV shows a high variation among individuals. The lack of a significant difference between the patient and control groups in the current study conducted with a limited number of patients may be due to these wide variations. Furthermore, changes in OBV can also be seen in certain common conditions such as respiratory infections and trauma. ​In a previous study, the OBV values ​​were reported to decrease following an acute event but there is an increase in the measurements undertaken after 1.5 years [25]. This confirms the high plasticity of OB.

Unlike OB, studies on the imaging of OT are limited in number. In the literature, there are postmortem, surgical, and radiological studies that evaluated OT. In a study measuring OTL in 10 cadavers, the mean OTL was reported as 30 mm [26]. In a surgical study, the mean OTL was measured as 29.3 mm [27]. The MRI imaging of OT was first introduced in a 1989 study, in which 65 patients with a corner tumor in the cerebellopontine angle and facial spasm were evaluated, and the mean OTL was reported as 25 mm. In the same study, it was noted that MRI examination was an appropriate method for visualizing OT, but it was difficult to describe this structure in axial sections [28]. In the current study, OTL measurements were easily performed on sagittal sections. In another study, 84% of 142 patients with complaints of headache, dizziness, and/or tinnitus, who had no olfactory dysfunction or the diagnosis of a neurodegenerative disease were visualized in axial and coronal sections [29].

In two different studies conducted with patients diagnosed with Parkinson’s disease, OT was examined using diffusion tensor MRI and microstructural damage was investigated. In both studies, the OLT of Parkinson’s patients was found to be significantly increased as compared to the control group, and microstructural damage was detected [30]. This suggests that OT can be structurally affected by neurodegeneration, which can be demonstrated radiologically. Starting from this point, we thought that OTL measurement might be an indicator of the structural effect on the olfactory system and undertook the current study in ET cases. In order to enrich our study, we found it appropriate to add OTL to OBV measurements to bring a different perspective to the evaluation of the olfactory system.

In this study, the OTL of each patient was measured in mm in sagittal sections. Both the right and left OTL values ​​were found to be significantly lower in the patient group as compared to the control group (p = 0.003, <0.001). This may be an anatomical indication of olfactory system involvement in ET patients. The current measurement method can be easily applied by doctors in clinical practice, and assessment only takes a short time. OTL measurement can be undertaken in many diseases encountered in clinical practice and may be a radiological indicator of neurodegeneration and thus assist in the evaluation of the olfactory system in these diseases. More significant findings can be obtained in wider-range studies with a larger number of patients. Our limitations include the small sample size and the absence of a smell function test. In addition, had the findings been confirmed by disease duration and severity, more significant results would have been achieved.

## Conclusions

The findings of our study support the idea that ET has a neurodegenerative nature. OTL measurement is an easy-to-use, non-invasive method that can be utilized in clinical practice as a radiological indicator of neurodegeneration. We consider that further multicenter, longitudinal studies with larger case series are needed in this area.
